# Evaluation of extraction methods for pharmacologically active compounds from anticonvulsant traditional Chinese medicines: Gou Teng, Tian Ma, Jiang Can using DART-TOF-MS

**DOI:** 10.1039/d0ay02015j

**Published:** 2021-01-18

**Authors:** Kimberly N. Karin, Justin L. Poklis, Michelle R. Peace

**Affiliations:** Department of Forensic Science, Virginia Commonwealth University Richmond VA USA mrpeace@vcu.edu; Department of Pharmacology and Toxicology, Virginia Commonwealth University Richmond VA USA

## Abstract

Chinese herbal medicines (CHMs) are classified as dietary supplements. Interactions with western medications, the presence of contaminants or adulterants, or a mis-labeled or mis-used CHM may lead to toxicological emergencies that can be undetected in death investigations. Laboratories must be able to efficiently analyze cases in which CHMs are suspected. Five extractions were evaluated for their ability to extract pharmacologically active compounds from herbal matrices: water, ethanol, microwave-assisted (MAE), ethanol : chloroform, and acid-wash. Anticonvulsive and other pharmacologically active compounds in Gou Teng, Tian Ma, and Jiang Can purchased from Beijing, China and New York were compared in the powder and the extracts using Direct Analysis in Real Time-Mass Spectrometry (DART-MS). Approximately 0.25 g of macerated herb was used per extraction. The water and ethanol extractions were simple liquid extractions. For the MAE, powdered herb was soaked in 65% ethanol, microwaved, and concentrated. The ethanol : chloroform extraction involved soaking in 1 : 1 ethanol : chloroform, sonication, and concentration. In the acid-wash extraction, powdered herb was soaked in acetic acid, followed by addition of sodium hydroxide, hexane extraction, and reconstitution in ethyl acetate. The powdered herbs and extracts were analyzed using a Jeol JMS T100LC AccuTOF DART-MS in positive and negative mode. Of the evaluated methods, no single extraction worked for all active compounds from the three CHMs. The MAE extract contained the most pharmacologically active compounds, while the acid-wash contained the least for the three products. Gou Teng purchased from different sources did exhibit a difference in pharmacologically active compounds, potentially from different species.

## Abstract 2

With the popularity of natural products, rapid analysis of pharmacologically active analytes from natural matrices is critical for public health. Microwave-assisted extraction with DART-MS provides efficient analysis of analytes from herbal matrices.

## Graphical abstract

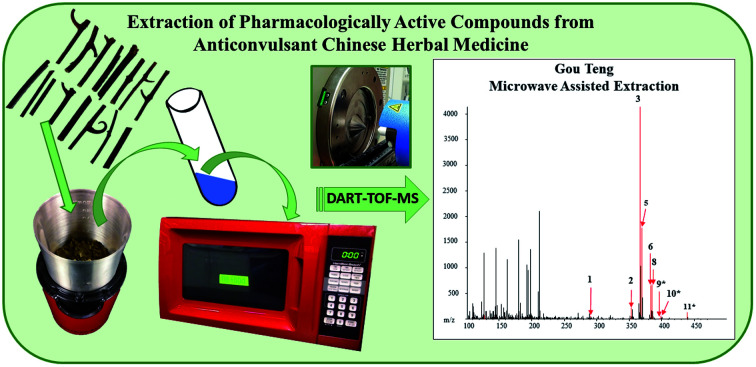

## 1. Introduction

Increasing global use of Chinese Herbal Medicines (CHM) is attributed to the availability, relatively low costs, and perceived lower risk of side effects from natural products compared to pharmaceuticals.^1^ With the approval by the World Health Organization (WHO) of the new version of the International Statistical Classification of Diseases and Related Health Problems (ICD), which includes a chapter on traditional Chinese medicine for the first time, the worldwide usage of traditional Chinese medicine is expected to continue to increase.^2^ In the United States alone, the sale of herbal dietary supplements exceeded 8 billion dollars in 2017.^3^ Consumers use herbal products, classified by their main action, for treatment of a wide variety of ailments. Anticonvulsants, such as Gou Teng, Tian Ma, and Jiang Can, are used primarily for the treatment of muscle spasms.^4^

Guo Teng, also known as cat's claw, is the dried stem from *Uncaria rhynchophylla*, *U. macrophylla*, *U. hissata*, *U. sessilifructus*, or *U. sinensis*.^5^ Beside the use of various parts of the plant being used to treat fever, dizziness, and spasms, they are also used as a sedative, analgesic, and antihypertensive.^4,5^ The main active alkaloids in Guo Teng (Table 1) include corynoxeine, isocorynoxeine, hirsuteine, hirsutine, rhynchophylline, and isorhynchophylline.^4^

**Table 1:** Reported biologically active compounds in the Traditional Chinese Medicine products: Gou Teng, Tian Ma, and Jiang Can and their monoisotopic mass. Bolded compounds are the reported main pharmacologically active compounds. Superscripts indicate referencesreporting the compound in the herbal product

Compound	Chemical formula	Mass	Compound	Chemical formula	Mass
**Gou Teng**					
Quinolic acid^5^	C_7_H_5_NO_4_	167.0219	**Corynoxeine** ^4,5,7^	C_22_H_26_N_2_O_4_	382.1893
Caffeic acid^5^	C_9_H_8_O_4_	180.0423	**Isocorynoxeine** ^5,7^		
Catechin^5^	C_15_H_14_O_6_	290.0790	Corynoxine B^5^	C_22_H_28_N_2_O_4_	384.2049
Epicatechin^5,6^			**Isorhynchophylline** ^4–7^		
Angustidine^5^	C_19_H_15_N_3_O	301.1215	**Rhynchophylline** ^4,5^		
Angustine^5^	C_20_H_15_N_3_O	313.1215	Campesterol^5^	C_28_H_48_O	400.3705
Angustoline^5^	C_20_H_17_N_3_O_2_	331.1321	Stigmasterol^5^	C_29_H_48_O	412.3705
Vallesiachotamine^5^	C_21_H_22_N_2_O_3_	350.1630	β-Sitosterol^5^	C_29_H_50_O	414.3862
Akuammigine^5^	C_21_H_24_N_2_O_3_	352.1787	Afzelin^6^	C_21_H_20_O_10_	432.1056
Tetrahydroalstonine^5^			Macrophylline A^5^	C_25_H_32_N_2_O_5_	440.2311
**Hirsuteine** ^4,5,7^	C_22_H_26_N_2_O_3_	366.1943	Quercitrin^6^	C_21_H_20_O_11_	448.1006
Isopteropodine^5^	C_21_H_24_N_2_O_4_	368.1736	Ursolic acid^6^	C_30_H_48_O_3_	456.3603
Mitraphylline^5^			Hyperin^4,6^	C_21_H_19_O_12_	463.0877
Pteropodine^5^			Strictosamide^5^	C_26_H_30_N_2_O_8_	498.2002
Dihydrocorynantheine^4,5,7^	C_22_H_28_N_2_O_3_	368.2100	Vincosamide^7^		
**Hirsutine** ^4,5^			3-α-Dihydrocadambine^5,7^	C_27_H_34_N_2_O_10_	546.2213
			Rutin^5^	C_27_H_30_O_16_	610.1534

**Tian Ma**					
4-Hydroxybenzaldehyde^8^	C_7_H_6_O_2_	122.0368	**Vanillyl alcohol** ^4^	C_8_H_10_O_3_	154.0630
4-Hydroxybenzyl alcohol^8,9^	C_7_H_8_O_2_	124.0524	Vanillic acid^8^	C_8_H_8_O_4_	168.0423
4-Hydroxybenzylmethylether^10^	C_8_H_10_O_2_	138.0681	**Gastrodin** ^4^	C_13_H_18_O_7_	286.1053
**Vanillin** ^4^	C_8_H_8_O_3_	152.0473			

**Jiang Can**					
**Ammonium oxalate** ^11^	C_2_H_8_N_2_O_4_	124.0484	Pinoresinol^11^	C_20_H_22_O_6_	358.1416
d-Mannitol^11^	C_6_H_14_O_6_	182.0790	**Aurantiamide** ^11^	C_25_H_26_N_2_O_3_	402.1943
Citric acid^11^	C_6_H_8_O_7_	192.0270	**β-Sitosterol** ^11^	C_29_H_50_O	414.3862
Kaempferol^11^	C_15_H_10_O_6_	286.0477	**Ergost-6,22-dien-3β, 5α, 8α-triol** ^11^	C_28_H_46_O_3_	430.3447
Quercetin^11^	C_15_H_10_O_7_	302.0427	**Beauvericin** ^11^	C_45_H_57_N_3_O_9_	783.4095

Tian Ma, from the dried tubes of *Gastrodia elata* Blume, is also known as Gastrodiae Rhizoma, Chi Jian, and Gui Du You^9^*.* Tian Ma can be used in the treatment of dizziness, headache, hypertension, and chest pain, as well as neurological disorders such as epilepsy, vertigo, and tetanus.^4,8^ The active components in Tian Ma (Table 1) include vanillin, vanillyl alcohol, and gastrodin.^4^

Jiang Can is a natural product composed of silkworm larva (*Bombyx mori* L.) killed and stiffened through infection by *Beauveria bassiana* forming a white, ammonium oxalate residue on the surface of the silkworm.^11,12^ Uses of Jiang Can include treatment of epilepsy, convulsions, cough, asthma, headaches, and postpartum pain. The reported active components in Jiang Can (Table 1) include ammonium oxalate, aurantiamide, beauvericin, ergost-6,22-dien-3β,5α,8α-triol, pinoresinol, and β-sitosterol.^4^

Although consumers associate lower risks of adverse effects with natural products, CHMs can pose a legitimate risk to consumers. CHMs are classified as dietary supplements which have a low standard for quality as defined by the U.S. Food and Drug Administration (FDA) in the Dietary Supplement Health and Education Act (DSHEA) of 1994.^13^ Regulatory standards for dietary supplements include that they cannot be unsanitary or toxic, or pose a significant risk to consumers.^13^ CHMs do not have a standardized naming convention, which can lead to potentially dangerous errors.^4^ And, potential negative interactions between multiple herbs or between herbs and over-the-counter and/or prescribed pharmaceutical products may not be considered or known when the herbs are consumed.^14^

Reported adverse drug reactions from herbal product use, whether it be from the use of an herbal product or a combination of herbal products are increasing.^15^ Annually China's drug regulator receives reports of more than 230 000 cases of adverse effects resulting from CHMs.^2^ Global use of CHMs is expected to continue to rise and with it potentially the occurrences of adverse reactions.^2^ Toxicological emergencies have been described in the literature, such as the case of a 36 year-old woman consuming an unknown herbal decoction for three days prior to admission to the hospital where she suffered cardiac arrest. Her father purchased the herbs based on the recommendation of a neighbor for the treatment of her aplastic anemia, but he did not know the name of the herbs.^16^ Another case involved the mistaken identity of a natural product, and a 66 year old man lost consciousness 10 minutes after consuming what he thought to be *Rhizopogon roseolus*.^17^ In cases such as these, it is critical to be able to rapidly identify pharmacologically active compounds in the herbal products. Adverse drug reactions and deaths related to consumption of herbal products are underreported because of the lack of testing. Due to the wide range of herbal products and pharmacologically active compounds in such products, a broad analytical scheme is advantageous for detection of these compounds. Current common methods for targeted identification of compounds in herbs include non-specific and/or time-consuming analytical methods such as thin layer chromatography (TLC) and high-performance liquid chromatography (HPLC).^1^

Extraction of the analytes of interest from herbal products can be complex and time consuming. Extraction methods can vary from liquid extraction followed by filtration or solid phase extraction to filtration with protein precipitation depending on the administration form of the CHM. A described method for *Fructus corni* required a 45 minute sonication in 80% methanol (v/v), dilution, and extraction using a C18 solid phase extraction column prior to ultra-fast liquid chromatography (UFLC) analysis.^18^ A 24 minute gradient was used to analyze the analytes of interest using UFLC. Other methods for separating the complex mixture of compounds using HPLC can take up to 65 minutes per sample.^19–22^

Particularly in the case of adverse drug reactions a rapid method requiring minimal sample preparation for the analysis of pharmacologically active compounds is advantageous. Direct Analysis in Real Time-Time of Flight mass spectrometry (DART-MS) is an atmospheric pressure ionization method with direct sampling to the mass spectrometer.^23^ DART-MS is a rapid technique with little to no sample preparation required.^23^ Pairing a quick, simple extraction method with DART-MS analysis could concentrate analytes of interest and remove compounds of non-interest, simplifying the mass spectra.

The aim of the study was to evaluate methods for analyte extraction paired with a rapid instrumental analysis, DART-MS, for pharmacologically active compounds in anticonvulsant herbal medicines, Gou Teng, Tian Ma, and Jiang Can. Different extraction methods were selected for evaluation based on being quick, simple methods that could be easily adopted by laboratories with the potential for extracting a wide variety of pharmacologically active compounds. The goal is for a broad extraction and analytical scheme with the ability to detect diverse pharmacologically active compounds from herbal matrices. Additionally, the herbal products were purchased from different locations with the purpose of comparing the anticonvulsive and pharmacologically active compounds in the herbs from different sources.

## 2. Experimental

### 2.1 Materials

Gou Teng, Tian Ma, and Jiang Can were purchased from Tong Ren Tang Chinese Medicine Company (Hong Kong, People's Republic of China) in Beijing, China and Chinatown New York. Tian Ma was also purchased from C.H.T. Inc. in Chinatown New York. The Gou Teng purchased in Chinatown New York was an Herbal Doctor product (Murray Int'l Trading Co., Inc., Brooklyn, NY) labeled as a product of China. The Jiang Can purchased in Chinatown New York was a Royal King product (Kwok Shing Hong, Inc., Brooklyn, NY) labeled as a product of the People's Republic of China. The Tian Ma purchased in Chinatown New York was packaged in a clear plastic bag with no product information.

Glacial acetic acid, sodium hydroxide (molecular biology grade), methanol, and *n*-hexane were purchased from Fisher Scientific (Hampton, NH). The ethyl acetate and dichloromethane used were from ACROS Organics (Geel, Belgium). Koptec 200 Proof Ethanol was purchased from Decon Labs Inc. (King of Prussia, PA). The chloroform used was from Pharmco-Aaper (Brookfield, CT). Polyethylene glycol (PEG) 600 was purchased from Ultra Inc. (North Kingstown, RI).

### 2.2 Sample preparation

The herb was ground into a powder using a Kitchen Aid blade coffee grinder (Benton Harbor, MI) and 0.25 g of powdered herbs was used for each extraction method. Five extractions: water, ethanol, microwave-assisted, ethanol : chloroform, and acid-wash were performed on the powdered herbs. The microwave-assisted, ethanol : chloroform, and acid-wash extractions were adapted from published methods.^24^

### 2.3 Extraction procedure

#### 2.3.1 Water extraction

One milliliter of warm deionized water was added to the powdered herb. The herb solution was heated at 50 °C on a hot plate for 20 minutes. Another 0.5 mL of hot water was added to the solution, since the herb soaked up the water. The solution was vortexed and then centrifuged at 3000 rpm for five minutes using an Eppendorf Centrifuge 5810R (Hauppauge, NY). The supernatant was removed and stored in the fridge.

#### 2.3.2 Ethanol extraction

The powdered herb was vortexed and soaked in one milliliter of 200 proof ethanol for four days. The solution was stored in the refrigerator prior to analysis by DART-MS.

#### 2.3.3 Microwave assisted extraction (MAE)

The powdered herb was added to 6 mL of 65% ethanol. The 65% ethanol herb solution was irradiated with 70 W (power level 1) using a Hamilton Beach 700W microwave (Glen Allen, VA) for one minute. The 6 mL extract was immediately concentrated to 1 mL using an Organomation N-Evap Analytical Evaporator (Berlin, MA).

#### 2.3.4 Ethanol : chloroform extraction (1 : 1 EtOH : CHCl_3_)

The powdered herb was soaked in 13 mL of 1 : 1 EtOH : CHCl_3_ overnight. The samples were sonicated for 30 min using a Fisherbrand CPX1800 Ultrasonic bath (Waltham, MA). The extract was concentrated to 1 mL using an Organomation N-Evap Analytical evaporator.

#### 2.3.5 Acid-wash

The powdered herb was soaked in 8 mL of glacial acetic acid overnight. One milliliter of 10 N sodium hydroxide was added to the acetic acid herb solution. An extraction using 2 mL of *n*-hexane was performed three times. The samples were dried and reconstituted in 1 mL of ethyl acetate.

### 2.4 DART-MS analysis

A previously validated method using a JEOL JMS T100LC Accu-TOF DART-MS (JEOL USA, Inc., Peabody, MA) was used in positive and negative mode to analyze the powdered herb and extracts.^25^ Briefly, orifice 1 was operated in function switching mode alternating between 20, 30, 60, and 90 V, while orifice 2 was operated at 5 V with a ring lens voltage of 3 V. The helium flow rate for the ion source was 2.0 L min^−1^ with a heater temperature of 350 °C. The following instrument parameters were used: discharge needle of 4000 V, detector voltage of 2000 V, and peaks voltage of 400 V (positive) or 800 V (negative). Electrode 1 and 2 were set at 150 V and 250 V, respectively. A mass range of 50 to 1500 *m*/*z* and 90 to 1500 *m*/*z* were scanned for positive and negative mode respectively.

A solution of PEG 600 in methanol (positive) or in 1 : 1 methanol : dichloromethane (negative) was used to calibrate the instrument and a positive control was used to confirm the mass values fall within ±5 mmu. For positive mode the positive control contained methamphetamine, cocaine, and nefazodone, while the negative mode control contained aspirin and furosemide.

At the beginning of each run the calibrator and positive control were wanded, followed by a blank. For the powdered herb a blank capillary tube served as the blank, while for the extracts a solvent blank was wanded using a capillary tube. Each sample was wanded five times using a capillary tube. Before the completion of the run, the positive control was run again to ensure mass accuracy over the course of the analysis.

The background subtracted mass spectra were analyzed and compared to a compiled list of compounds referenced in literature as being contained in the herb. Data analysis was performed using T.S.S Pro version 3.0 and Mass Mountaineer. For an identification of a compound in the herb product, the mass from the spectrum was required to be within a ±5 mmu range from the monoisotopic mass of the compound.

## 3. Results & discussion

### 3.1 Gou Teng

The DART-MS spectra and the pharmacologically active compounds for the powdered Gou Teng from Tong Ren Tang in Beijing and New York were compared. Out of the three products, Gou Teng was the only product to contain a difference in pharmacologically active compounds between the products purchased from different locations. Gou Teng purchased from Beijing contained the isobaric compounds, dihydrocorynantheine/hirsutine ([M + H]^+^ = 369.2178), while Gou Teng purchased from New York contained the isobaric compounds, isopteropodine/mitraphylline/pteropodine ([M + H]^+^ = 369.1814). Differences in pharmacologically active compounds in the herbal product could result from the use of different species. *Uncaria rhynchophylla*, *U. macrophylla*, *U. hissata*, *U. sessilifructus*, or *U. sinensis* are all common species marketed under Gou Teng.^4^ The product purchased in New York was labeled as *U. rhynchophylla*, while the product purchased from Beijing had no indication of species on the packaging.

**Fig. 1:**
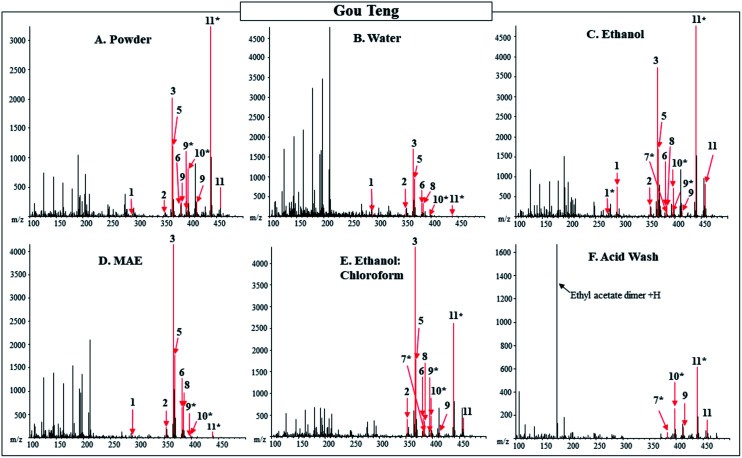
DART-MS (20 V) positive ionization mode spectra of powdered and extracts of Gou Teng from Tong Ren Tang purchased in Beijing. The powdered material is shown in Panel (A) compared to water, ethanol, microwave-assisted, ethanol : chloroform, and acid wash extracts depicted in Panel (B–F) respectively. Labeled numbers correspond to pharmacologically active compounds found in Table 2. Gou Teng purchased from Beijing (depicted in figure) contained the isobaric compounds, dihydrocorynantheine/hirsutine ([M + H]^+^ = 369.2178), while Gou Teng purchased from New York contained the isobaric compounds, isopteropodine/mitraphylline/pteropodine ([M + H]^+^ = 369.1814). * designates M − OH adduct.

The pharmacologically active compounds detected in the extracts from the five different extractions were compared to those detected in the powdered Gou Teng (Table 2 and Fig. 1). Of the 24 compounds with unique masses searched for in Gou Teng, 11 were detected between the powder and the extracts. For the extractions, the ethanol extract was the only one to contain all the pharmacologically active compounds detected in the powder. Additionally, campesterol was detected in the ethanol extract, but not the powdered product. The reported main pharmacologically active compounds: corynoxeine, isocorynoxeine, hirsuteine, hirsutine, isorhynchophylline, and rhynchophylline were all detected in the following extracts: water, ethanol, MAE, and ethanol : chloroform, in addition to the powdered product. No additional pharmacologically active compounds were detected in negative mode for the powdered product or extracts of Gou Teng.

**Table 2:** Summary of pharmacologically active compounds from Gou Teng detected in 20 V DART-MS analysis of powdered product and extracts and corresponding reported biological activity of the compounds. X designates compounds detected in all samples, while B indicates the compound was only detected in products purchased in Beijing, or NY for products purchased in New York. * designates M − OH adduct. Bolded compounds are reported main pharmacologically active compounds

Gou Teng										
#	Compound	Biological activity	Adduct	Adduct mass	Extraction method					
					Powder	H_2_O	EtOH	MAE	EtOH : CHCl_3_	Acid wash
1*	Catechin	Antioxidant^26^	–OH	273.0763			X			
	Epicatechin	Antioxidant^26^	–OH							
1	Catechin	Antioxidant^26^	+H	291.0869	X	X	X	X		
	Epicatechin	Antioxidant^26^	+H							
2	Akuammigine	Weak antagonist of pre & postsynaptic α-adrenoceptors^27^	+H	353.1865	X	X	X	X	X	
	Tetrahydroalstonine	Antagonist of pre α2-adrenoceptors^27^	+H							
3	**Hirsuteine**	5-HT3 antagonist^28^	+H	367.2022	X	X	X	X	X	
4	Isopteropodine	Modulate function of G protein-coupled muscarinic M(1) acetylcholine & 5-HT(2) receptors^29^	+H	369.1814	NY	NY	NY	NY	NY	
	Mitraphylline	Anti-inflammatory^30^	+H							
	Pteropodine	Modulate function of G protein-coupled muscarinic M(1) acetylcholine & 5-HT(2) receptors^29^	+H							
5	Dihydrocorynantheine	Vasodilator^5^	+H	369.2178	B	B	B	B	B	
	**Hirsutine**	5-HT3 antagonist^28^	+H							
6	**Corynoxeine**	5-HT3 antagonist^28^	+H	383.1971	X	X	X	X	X	
	**Isocorynoxeine**	5-HT3 antagonist^28^	+H							
7*	Campesterol	Inhibits cholesterol absorption^31^	–OH	383.3678			X		X	X
8	Corynoxine B	Inhibitor of central dopamine release^32^	+H	385.2127	X	X	X	X	X	
	**Isorhynchophylline**	5-HT3 antagonist,^28^ antagonist of NMDA-type ionotropic glutamate receptor^33^	+H							
	**Rhynchophylline**	5-HT3 antagonist (Nakamura), antagonist of NMDA-type ionotropic glutamate receptor^32^	+H							
9*	Stigmasterol	Inhibits cholesterol absorption^31^	–OH	395.3678	X		X	X	X	
10*	β-Sitosterol	Inhibits cholesterol absorption^31^	–OH	397.3834	X	X	X	X	X	X
9	Stigmasterol	Inhibits cholesterol absorption^31^	+H	413.3783	X		X		X	X
11*	Ursolic acid	Anti-inflammatory, Antihyperlipidemic^34^	–OH	439.3576	X	X	X	X	X	X
11	Ursolic acid		+H	457.3682	X		X		X	X

Although not all reported pharmacologically active compounds were detected in all the products, compounds were detected in each of the products that may be responsible for producing the reported effects. The main pharmacologically active compounds: corynoxeine, isocorynoxeine, hirsuteine, hirsutine, isorhynchophylline, and rhynchophylline are reported 5-HT_3_ antagonists.^28^ Conflicting research debating whether antagonism of the 5-HT_3_ serotonergic receptor reduces seizures and convulsions, with reports of convulsions being decreased and other reports of increased convulsions by the administration of a 5-HT_3_ antagonist.^35^ Additionally, isorhynchophylline is a reported antagonist of *N*-methyl-d-aspartate (NMDA)-type ionotropic glutamate receptors.^9^ Antagonism of the NMDA receptors has reported anticonvulsant effects and is a therapeutic target for antiepileptic treatments.^36^ The reported main pharmacologically active compounds of Gou Teng detected in the powder and multiple extracts could produce the reported anticonvulsant effects.

**Fig. 2:**
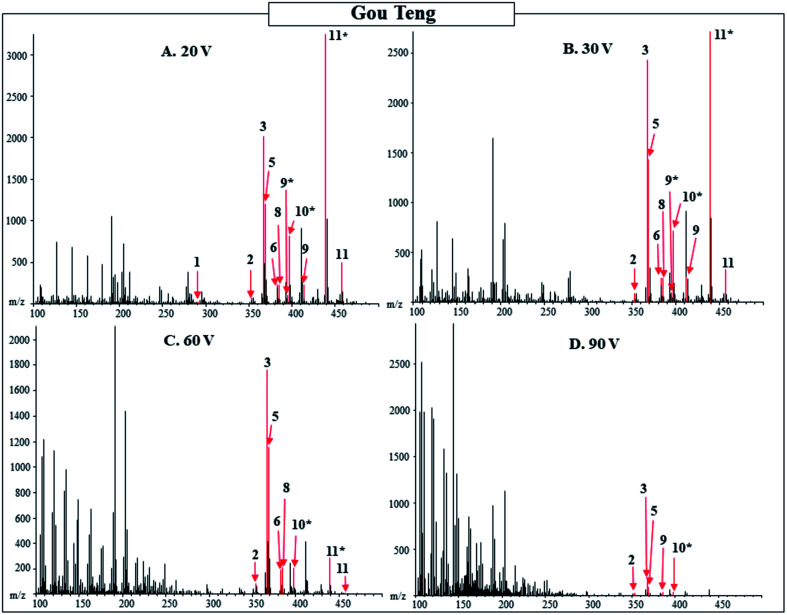
Function-switching DART-MS spectra of powdered Gou Teng from Tong Ren Tang purchased in Beijing. Orifice 1 voltage was alternated between 20, 30, 60, and 90 V and the corresponding spectra are shown in Panels (A)–(D) respectively. Labeled numbers correspond to pharmacologically active compounds found in Table 3. * designates M − OH adduct.

Additionally, the 20, 30, 60, and 90 V spectra were compared for the powdered herbal products with results from Gou Teng shown as an example in Table 3 and Fig. 2. The higher orifice voltage spectra were compared to the 20 V spectra for the powdered product. As the voltage increased, the less intense [M + H]^+^ or [M − OH]^−^ peaks were no longer detected, while the more intense peaks were still observed in the 30, 60, and 90 V spectra. The higher molecular weight compounds fragmented and were no longer observed in the higher voltage spectra. While fragmentation patterns of compounds would provide additional confirmation of the pharmacologically active compounds, when a technique such as DART-MS, with no separation of compounds, is used the interpretation of the fragmentation is complicated for a complex mixture. Additionally, standards for all pharmacologically active compounds in the herbal products are not available. Individual standards of the pharmacologically active compounds would supply additional evidence for the identification of the pharmacologically active compounds based on fragmentation pattern. A separation technique or standards would be required to determine the fragmentation patterns of the compounds, without either of those the 30, 60, and 90 V spectra do not provide additional information for the identification of pharmacologically active compounds present.

**Table 3:** Comparison of pharmacologically active compounds detected in Gou Teng in 20, 30, 60, 90 V DART-MS spectra. * designates M − OH adduct. Bolded compounds are reported main pharmacologically active compounds

Gou Teng							
#	Compound	Adduct	Adduct mass	Orifice 1 voltage			
				20 V	30 V	60 V	90 V
1*	Catechin	–OH	273.0763				
	Epicatechin	–OH					
1	Catechin	+H	291.0869	X			
	Epicatechin	+H					
2	Akuammigine	+H	353.1865	X	X	X	X
	Tetrahydroalstonine	+H					
3	**Hirsuteine**	+H	367.2022	X	X	X	X
4	Isomitraphylline	+H	369.1814	X	X	X	X
	Isopteropodine	+H					
	Mitraphylline	+H					
	Pteropodine	+H					
5	Dihydrocorynantheine	+H	369.2178	X	X	X	X
	**Hirsutine**	+H					
6	**Corynoxeine**	+H	383.1971	X	X	X	
	**Isocorynoxeine**	+H					
7*	Campesterol	–OH	383.3678				
8	Corynoxine B	+H	385.2127	X	X	X	X
	**Isorhynchophylline**	+H					
	**Rhynchophylline**	+H					
9*	Stigmasterol	–OH	395.3678	X	X		
10*	β-Sitosterol	–OH	397.3834	X	X	X	X
9	Stigmasterol	+H	413.3783	X	X		
11*	Ursolic acid	–OH	439.3576	X	X	X	
11	Ursolic acid	+H	457.3682	X	X	X	

### 3.2 Tian Ma

**Fig. 3:**
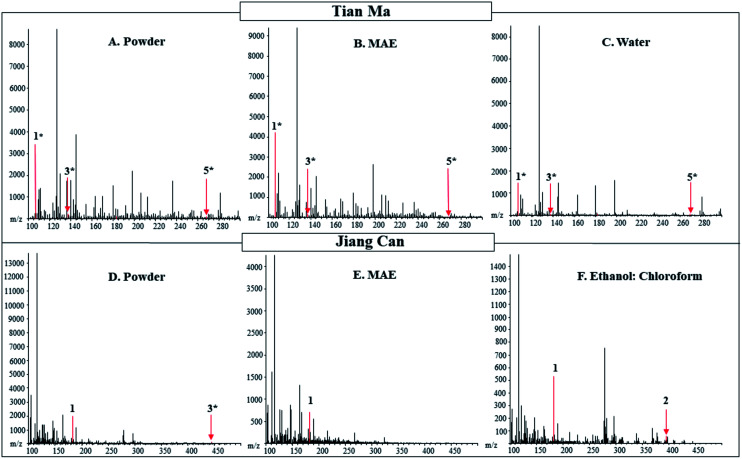
DART-MS (20 V) positive ionization mode spectra of powdered and extracts of Tian Ma from C.H.T. Inc. and Jiang Can from Tong Ren Tang purchased in New York. The powdered material of Tian Ma and Jiang Can is shown in Panels (A and D), respectively. The microwave-assisted extract is shown in Panel (B) for Tian Ma and (E) for Jiang Can. In Panel (C) is the water extract of Tian Ma and in Panel (F) is the ethanol : chloroform extract of Jiang Can. Labeled numbers correspond to pharmacologically active compounds found in Table 4. * designates M − OH adduct.

The pharmacologically active compounds detected in the extracts from the five different extractions were compared to those detected in the powdered Tian Ma (Table 4 and Fig. 3). For Tian Ma, seven pharmacologically active compounds with unique masses were searched for in the powder and the extracts. Of the seven, five unique masses were detected in the powder with no additional masses detected in the extracts. The following compounds were detected in the powdered Tian Ma: 4-hydroxybenzyl alcohol, vanillin, vanillyl alcohol, vanillic acid, and gastrodin. Vanillin and vanillic acid were only detected in negative mode. No single extract contained all the pharmacologically active compounds detected in the powder, but the MAE extract contained the most pharmacologically active compounds. The acid wash extraction was not effective in extracting any target pharmacologically active compounds from Tian Ma.

**Table 4:** Summary of pharmacologically active compounds from Tian Ma and Jiang Can detected in 20 V DART-MS analysis of powdered product and extracts and corresponding reported biological activity of the compounds. [–H] adducts were detected in negative ionization mode. * designates M − OH adduct. Bolded compounds are reported main pharmacologically active compounds

#	Compound	Biological activity	Adduct	Adduct mass	Extraction method					
					Powder	H_2_O	EtOH	MAE	EtOH : CHCl_3_	Acid wash
**Tian Ma**										
1*	4-Hydroxybenzyl alcohol	Suppress dopaminergic & serotonergic activity^37^	–OH	107.0497	X	X	X	X	X	
2	**Vanillin**	Anti-inflammatory^8^	–H	151.0395	X		X			
3*	**Vanillyl alcohol**	Suppress oxidative stress^38^	–OH	137.0603	X	X	X	X		
4	Vanillic acid	Inhibits inflammatory pain^39^	–H	167.0344	X			X		
5*	**Gastrodin**	Anti-depressant^40^	–OH	269.1025	X	X		X		

**Jiang Can**										
1	d-Mannitol	Diuretic^41^	+H	183.0869	X	X	X	X	X	
2	Citric acid	Antioxidant^42^	–H	191.0192	X	X		X		X
3*	**β-Sitosterol**	Anticonvulsant^11^	–OH	397.3834					X	X

In Tian Ma, all the reported main pharmacologically active compounds: 4-hydroxybenzaldehyde, gastrodin, vanillin, and vanillyl alcohol were detected in the powdered Tian Ma. 4-Hydroxybenzaldehyde is a reported GABA_A_ (γ-aminobutyric acid) receptor chloride channel complex agonist.^37^ The reported anticonvulsant effects of Tian Ma could be a result of the 4-hydroxybenzaldehyde through agonism of the GABA_A_ receptor, producing an inhibitory effect.^43^

### 3.3 Jiang Can

The pharmacologically active compounds detected in the extracts from the five different extractions were compared to those detected in the powdered Jiang Can (Table 4 and Fig. 3). For Jiang Can, ten pharmacologically active compounds with uniques masses were searched for in the powder and the extract. Between the powdered Jiang Can and the extracts ammonium oxalate, d-mannitol, citric acid, and β-sitosterol were detected. The ethanol extract contained only one pharmacologically active compound from Jiang Can, while the rest of the extracts contained two pharmacologically active compounds. d-Mannitol was detected in each extract except the acid wash extract. The ethanol : chloroform and acid wash extracts were the only extracts to contain an anticonvulsive compound, β-sitosterol. Citric acid was only detected in negative mode and was detected in the water and MAE extracts, in addition to the powder.

Even though a visible white residue associated with ammonium oxalate was present on both the Jiang Can products, the parent compound was not detected. Upon heating, a proton from the ammonium can transfer to the base producing ammonia gas and the conjugate acid of the salt.^44^ Heat from the helium stream could cause the proton transfer from the ammonium to the oxalate producing ammonia gas and the oxalate conjugate acid. A peak with a *m*/*z* of 178.9828 in the negative spectra of Jiang Can powder could correlate to the dimer of the oxalate conjugate acid.

Of the compounds detected, ammonium oxalate and β-sitosterol have reported antiepileptic and anticonvulsant activities.^11^ Although not all the reported main pharmacologically active compounds were detected in the powder nor the extracts, the ammonium oxalate and β-sitosterol could produce the reported anticonvulsant effects of Jiang Can.

The MAE was the most effective extraction for pharmacologically active compounds from these anticonvulsant herbs. Despite the herbs soaking in the extraction solvents longer in the ethanol, ethanol : chloroform, and the acid wash extractions than in the MAE, they were less effective in extracting the pharmacologically active compounds. Shorter extraction times can result in some active compounds being below the limit of detection. Not only was the MAE most effective at extracting these structurally diverse compounds, the MAE requires the least amount of time for extraction.

In general, for compounds not detected in the extracts nor the powder of these anticonvulsant products it is possible the compounds may be present below the limit of detection or the compounds may only be present in select species and not in the purchased products. Compounds detected in the extracts but not in the powder may be present below the limit of detection and were concentrated through the extraction process. Of the compounds detected in the powder, but not the extracts, many are less polar compounds with potentially poor extraction efficiency in more polar solvents used in the methods. Although not all pharmacologically active compounds were extracted, a wide variety of the structurally diverse active compounds were identified in the extracts.

With increasing popularity of natural products and the risk of toxicological emergencies and deaths from their use it is critical to be able to analyze a broad range of natural products. The two previously described cases in which the woman consumed an unknown combination of herbs and the 66 year old man consumed what he believed to be *Rhizopogon roseolus* when he actually ingested *Schleroderma albidum*, exemplify medically and forensically relevant cases in which an unknown product was consumed and an analysis is tedious and long. A rapid, inclusive extraction and analytical scheme could prove useful in identifying the products.^16^

## 4. Conclusions

Out of the five extraction methods evaluated, no single extraction worked for all the anticonvulsant and pharmacologically active compounds from the three different herbal products. The MAE extract contained the most pharmacologically active compounds, while the acid wash contained the least from the three products. While the ethanol extraction was the most effective extraction for Gou Teng, the MAE extraction was most effective for Tian Ma. For Jiang Can, no extraction clearly out-performed the others at extracting pharmacologically active compounds, but the ethanol : chloroform and acid wash extracts were the only extracts to contain an anticonvulsive compound. The pharmacologically active compounds, particularly the anticonvulsive compounds, from each product are structurally diverse so not one method will extract all compounds and the extraction method will determine the pharmacologically active compounds detected. Although no one method can extract all the pharmacologically active compounds, all the methods evaluated are easily adoptable, rapid extraction methods with common solvents such that products can be concurrently extracted for analysis. The DART-MS proved to be a quick and efficient analytical tool, such that multiple extractions could be easily assessed in sequence. With the growing popularity of natural products, rapid extraction and the efficient analysis of pharmacologically active compounds from natural matrices is critical for public health and public safety.

## Conflicts of interest

There are no conflicts to declare.

## Supplementary Material
